# Supplementary material for: Identification and Quantification of Urinary Microbial Phenolic Metabolites by HPLC-ESI-LTQ-Orbitrap-HRMS and Their Relationship with Dietary Polyphenols in Adolescents
Source: Antioxidants (Basel). 2022 Jun 14;11(6):1167. doi: 10.3390/antiox11061167 (PMC9220091; doi:10.3390/antiox11061167)
Supplement: Supplementary file 1 [file antioxidants-11-01167-s001.zip › antioxidants-1723878-supplementary.pdf]

Identification and quantification of urinary microbial phenolic metabolites by HPLC-ESI-LTQ-Orbitrap-HRMS and their relationship with dietary polyphenols in adolescents.

Supplementary Data

Table of contents

- **Standards and chemicals:** Commercial sources of standards and chemicals employed in the study.
- **Analytical condition testing before validation HPLC/ESI-LTQ-Orbitrap-HRMS method**

- **Figure S1.** Recovery obtained according to different solid phase extraction and reverse-phase chromatographic columns (Kinetex F5 (50 x 4.6 mm i.d., 2.6 μm) and Atlantis T3 C18 (100 x 2.1 mm i.d., 3 μm).
- **Figure S2.** Postpreparative stability. Mean concentrations (μg/L) of phenolic compounds recovered at the start (t = 0) and at 24 h with two standard concentrations prepared in synthetic urine.
- **Figure S3.** General characteristics of participants according to gender.
- **Figure S4.** Principal component (PC) biplot of subclass of microbial phenolic metabolites (MPM) according to tertiles of total polyphenol intake (*n*=546).
- **Figure S5.** Heatmap of the Pearson correlation between subclass of microbial phenolic metabolites and polyphenol-rich food intake in adolescents.
- **Table S1.** Recovery obtained in Oasis HLB and HLB prime.
- **Table S2.** Validation data: Linearity ranges, coefficient of determination, and low limits of detection and quantification of microbial phenolic metabolites.
- **Table S3.** Intra- and inter-day precision and accuracy, matrix effect and recovery results for three concentration levels (high, medium, and low); RSD (%) was calculated for the recovery values for three replicates.
- **Table S4**. Identification of microbial phenolic metabolites in urine by HPLC/ESI-LTQ-Orbitrap-HRMS.
- **Table S5**. Pearson correlation coefficients between microbial phenolic metabolites and dietary polyphenols in adolescents.
- **References**

Standards and chemicals

Gallic acid, 3-hydroxytyrosol, protocatechuic acid, 4-hydroxybenzoic acid, 3,4-dihydroxyphenylpropionic acid, 3′-hydroxyphenylacetic acid, *o*-coumaric acid, *m*-coumaric acid, *p*-coumaric acid, enterodiol, urolithin-A, and urolithin-B were purchased from Sigma-Aldrich (St. Louis, MO, USA). 3′-hydroxytyrosol-3′-glucuronide, dihydroresveratrol, and (+)*cis, trans*-abscisic acid D_6 were obtained from Santa Cruz (Santa Cruz Biotechnology, Santa Cruz, CA). 3-hydroxybenzoic acid, vanillic acid, syringic acid, enterolactone, and creatinine were purchased from Fluka (St. Louis, MO, USA). Standards were stored in powder form and protected from light. Methanol (MeOH) of LC-MS and acetonitrile (MeCN) of HPLC grade were obtained from Sigma-Aldrich (St. Louis, MO, USA), formic acid (≥ 98 %) from Panreac Química S.A. (Barcelona, Spain), and ultrapure water (Milli-Q) from a Millipore system (Bedford, USA).

Analytical condition testing before validation HPLC/ESI-LTQ-Orbitrap-HRMS method

- **Solid phase extraction (SPE) cartridge selection**

  Two different chemical cartridges, Waters Oasis HBL (hydrophilic-lipophilic-balance cartridge) 96-well plates 30 µm (30 mg) and Oasis PRiME HLB 96-well plates 3 mg, for the extraction of 3-hydroxybenzoic acid, 3-hydroxytyrosol, 3′-hydroxytyrosol-3′-glucuronide, protocatechuic acid, 4-hydroxybenzoic acid, vanillic acid, syringic acid, enterodiol, enterolactone, urolithin-B, gallic acid, dihydroresveratrol, urolithin-A, 3,4-dihydroxyphenylpropionic acid, 3′-hydroxyphenylacetic acid, *o*-coumaric acid, *m*-coumaric acid, and *p*-coumaric acid. Synthetic urine was spiked with 1000 µg/L of phenolic standards, and 500 µg/L of internal standard. Oasis HLB procedure was performed using the method previously describe by Martínez-Huélamo et al. for the analysis of polyphenols and their metabolites in urine samples, with some modifications [1]. Briefly, cartridges were activated with 1 mL of methanol (MeOH) and 1 mL of 0.1% formic acid. After loading 1 mL of acidified sample, clean-up was performed with 1 mL of 0.1% formic acid and 0.5% MeOH, and the elution with 1 mL MeOH acidified with 0.1% of formic acid. The eluted fraction was evaporated to dryness under a stream of nitrogen and reconstituted with 100 µL of 0.1% formic acid in water. To assess extraction recovery, blank synthetic urine extract spiked after SPE were also prepared at the same concentration (Table S1).

- **Optimization of SPE using different solutions**

  After the selection of Waters Oasis HLB 96-well plates 30 µm (30 mg) as cartridge for the SPE, four different SPE procedures were tested to obtain higher extraction of 3-hydroxybenzoic acid, 3-hydroxytyrosol, 3′-hydroxytyrosol-3′-glucuronide, protocatechuic acid, 4-hydroxybenzoic acid, vanillic acid, syringic acid, enterodiol, enterolactone, urolithin-B, gallic acid, dihydroresveratrol, urolithin-A, 3,4-dihydroxyphenylpropionic acid, 3′-hydroxyphenylacetic acid, *o*-coumaric acid, *m*-coumaric acid, and *p*-coumaric acid.

  Synthetic urine was spiked with 400 µg/L of phenolic standards and internal standard. These procedures were performed using the methods previously described by Martínez-Huélamo et al. and Quifer-Rada [1–3], with some modifications, following the next extractions:

  - *SPE 1:* cartridges were activated with 1 mL of methanol (MeOH) and 1 mL of 0.1% formic acid. After loading 1 mL of acidified sample, clean-up was performed with 1 mL of 0.1% formic acid and 0.5% MeOH, and the elution with 1 mL MeOH acidified with 0.1% of formic acid. The eluted fraction was evaporated to dryness under a stream of nitrogen and reconstituted with 100 µL of 0.05% formic acid in water.
  - *SPE 2:* cartridges were activated with 1 mL of methanol (MeOH) and 1 mL of 1.5 M formic acid. After loading 1 mL of sample, clean-up was performed with 0.5 mL of 1.5 M formic acid and 0.5% MeOH, and the elution with 1 mL MeOH acidified with 0.1% of formic acid. The eluted fraction was evaporated to dryness under a stream of nitrogen and reconstituted with 100 µL of 0.05% formic acid in water.
  - *SPE 3:* cartridges were activated with 1 mL of methanol (MeOH) and 1 mL of 1.5 M formic acid. After loading 1 mL of sample, clean-up was performed with 0.5 mL of 1.5 M formic acid and 0.5 mL of 5% MeOH, and the elution with 1 mL MeOH acidified with 0.1% of formic acid. The eluted fraction was evaporated to dryness under a stream of nitrogen and reconstituted with 100 µL of 0.05% formic acid in water.
  - *SPE 4:* cartridges were activated with 1 mL of methanol (MeOH) and 1 mL of 1% formic acid. After loading 1 mL of acidified sample, clean-up was performed with 1 mL of 1% formic acid and 2% MeOH, and the elution with 1 mL MeOH acidified with 0.1% of formic acid. The eluted fraction was evaporated to dryness under a stream of nitrogen and reconstituted with 100 µL of 0.05% formic acid in water.

- **Chromatographic column selection**

  Two different reverse-phase chromatographic columns were tested: Kinetex F5 (50 x 4.6 mm i.d., 2.6 µm) (Phenomenex, Torrance, CA, USA) and Atlantis T3 C18 (100 x 2.1 mm i.d., 3 µm) (Waters, Milford, MA, USA) (Figure S1).

Figures

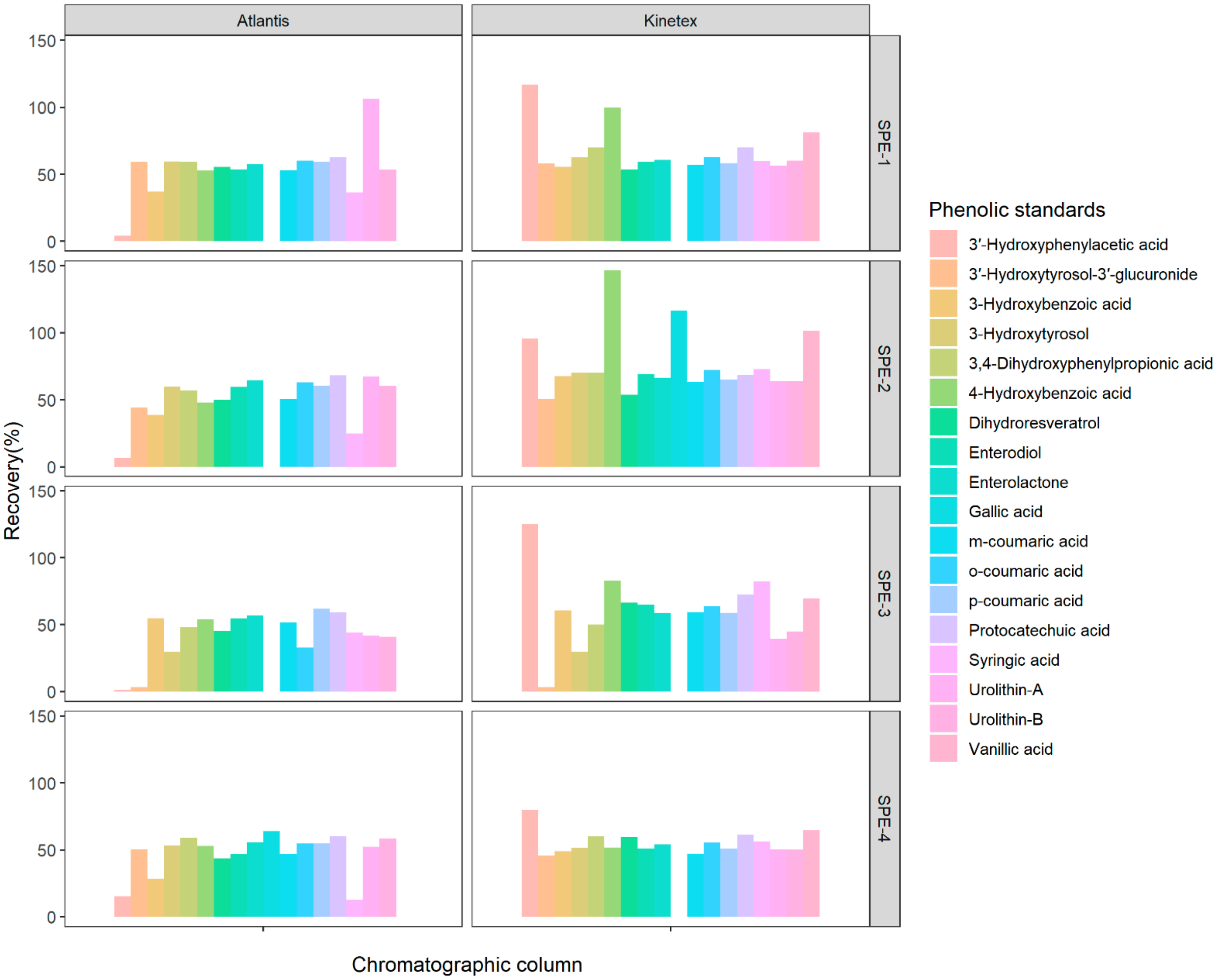

Figure S1. Recovery obtained according to different solid phase extraction and reverse-phase chromatographic columns (Kinetex F5 (50 x 4.6 mm i.d., 2.6 µm) and Atlantis T3 C18 (100 x 2.1 mm i.d., 3 µm).

SPE-1: cartridges were activated with 1 mL of methanol (MeOH) and 1 mL of 0.1% formic acid. After loading 1 mL of acidified sample, clean-up was performed with 1 mL of 0.1% formic acid and 0.5% MeOH, and the elution with 1 mL MeOH acidified with 0.1% of formic acid. The eluted fraction was evaporated to dryness under a stream of nitrogen and reconstituted with 100 µL of 0.05% formic acid in water.

SPE-2: cartridges were activated with 1 mL of methanol (MeOH) and 1 mL of 1.5 M formic acid. After loading 1 mL of sample, clean-up was performed with 0.5 mL of 1.5 M formic acid and 0.5% MeOH, and the elution with 1 mL MeOH acidified with 0.1% of formic acid. The eluted fraction was evaporated to dryness under a stream of nitrogen and reconstituted with 100 µL of 0.05% formic acid in water.

SPE-3: cartridges were activated with 1 mL of methanol (MeOH) and 1 mL of 1.5 M formic acid. After loading 1 mL of sample, clean-up was performed with 0.5 mL of 1.5 M formic acid and 0.5 mL of 5% MeOH, and the elution with 1 mL MeOH acidified with

0.1% of formic acid. The eluted fraction was evaporated to dryness under a stream of nitrogen and reconstituted with 100 μL of 0.05% formic acid in water.

SPE-4: cartridges were activated with 1 mL of methanol (MeOH) and 1 mL of 1% formic acid. After loading 1 mL of acidified sample, clean-up was performed with 1 mL of 1% formic acid and 2% MeOH, and the elution with 1 mL MeOH acidified with 0.1% of formic acid. The eluted fraction was evaporated to dryness under a stream of nitrogen and reconstituted with 100 μL of 0.05% formic acid in water.

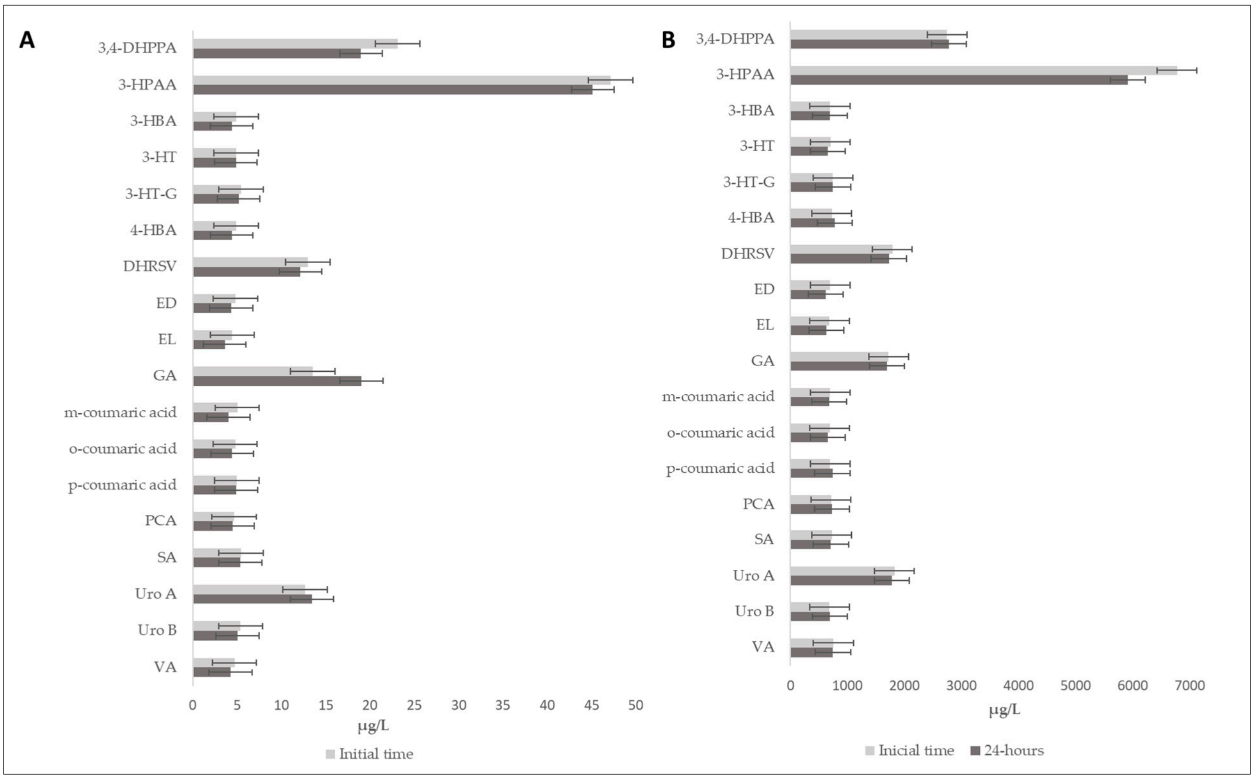

Figure S2. Postpreparative stability. Mean concentrations (μg/L) and SEM of phenolic compounds recovered at 0 h (t = 0) and at 24 h with two standard concentrations prepared in synthetic urine. **A**: stability of the low concentration *(5 μg L^{-} for 3-HBA, 3-HT, 3-HT-G, PCA, 4-HBA, VA, SA, p,m,o-coumaric acids, ED, EL, Uro B; 12.5μg L^{-1} for GA, DHRSV, and Uro A; 25 μg L^{-1} for 3,4-DHPPA, 50 μg L^{-1} for 3-HPAA).* **B**: stability of the high concentration *(766 μg L^{-} for 3-HBA, 3-HT, 3-HT-G, PCA, 4-HBA, VA, SA, p,m,o-coumaric acids, ED, EL, Uro B; 1915μg L^{-1} for GA, DHRSV, and Uro A; 3830 μg L^{-1} for 3,4-DHPPA, 7660 μg L^{-1} for 3-HPAA).* 3,4-DHPPA 3,4-dihydroxyphenylpropionic acid, 3-HPAA 3-hydroxyphenylacetic acid, 3-HBA 3-hydroxybenzoic acid, 3-HT 3-hydroxytyrosol, 3-HT-G 3-hydroxytyrosol glucuronide, 4-HBA 4-hydroxybenzoic acid, DHRSV dihydroresveratrol, ED enterodiol, EL enterolactone, GA gallic acid, PCA protocatechuic acid, SA syringic acid, Uro A urolithin A, Uro B urilithin B, VA vanillic acid.

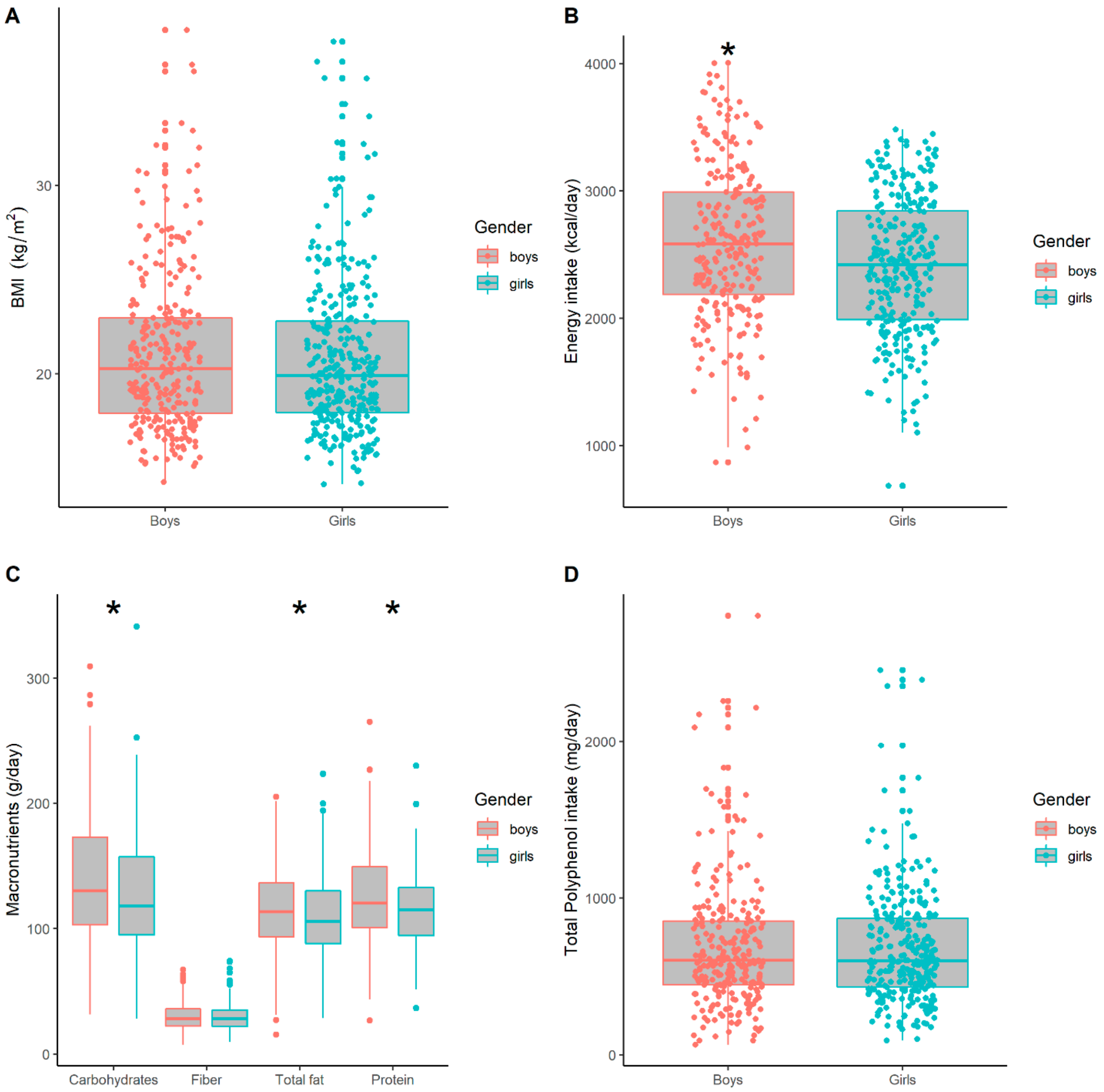

Figure S3. Boxplot of general characteristics of participants according to gender.

BMI body mass index.**A**: BMI (kg/m²), **B**: Total Energy Intake (kcal/day), **C**: Macronutrients (g/day), **D**: Total polyphenol intake (mg/day).* *p*-values < 0.05 from T-test analysis.

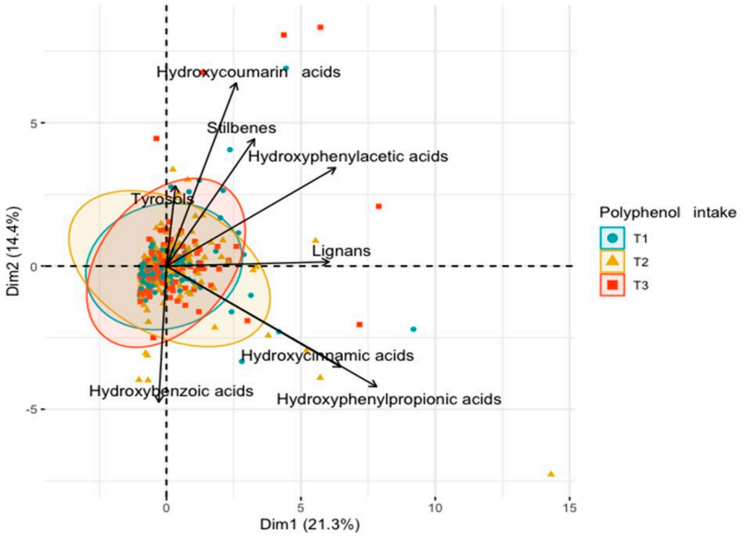

Figure S4. Principal Component (PC) biplot of subclass of microbial phenolic metabolites (MPM) according to tertiles of total polyphenol intake (*n*=546).

PC1, accounting for 21.33% of the total variance, included lignans; and PC2, accounting for 14.40%, included hydroxycoumarin acids, hydroxybenzoic acids, tyrosols, and stilbenes.

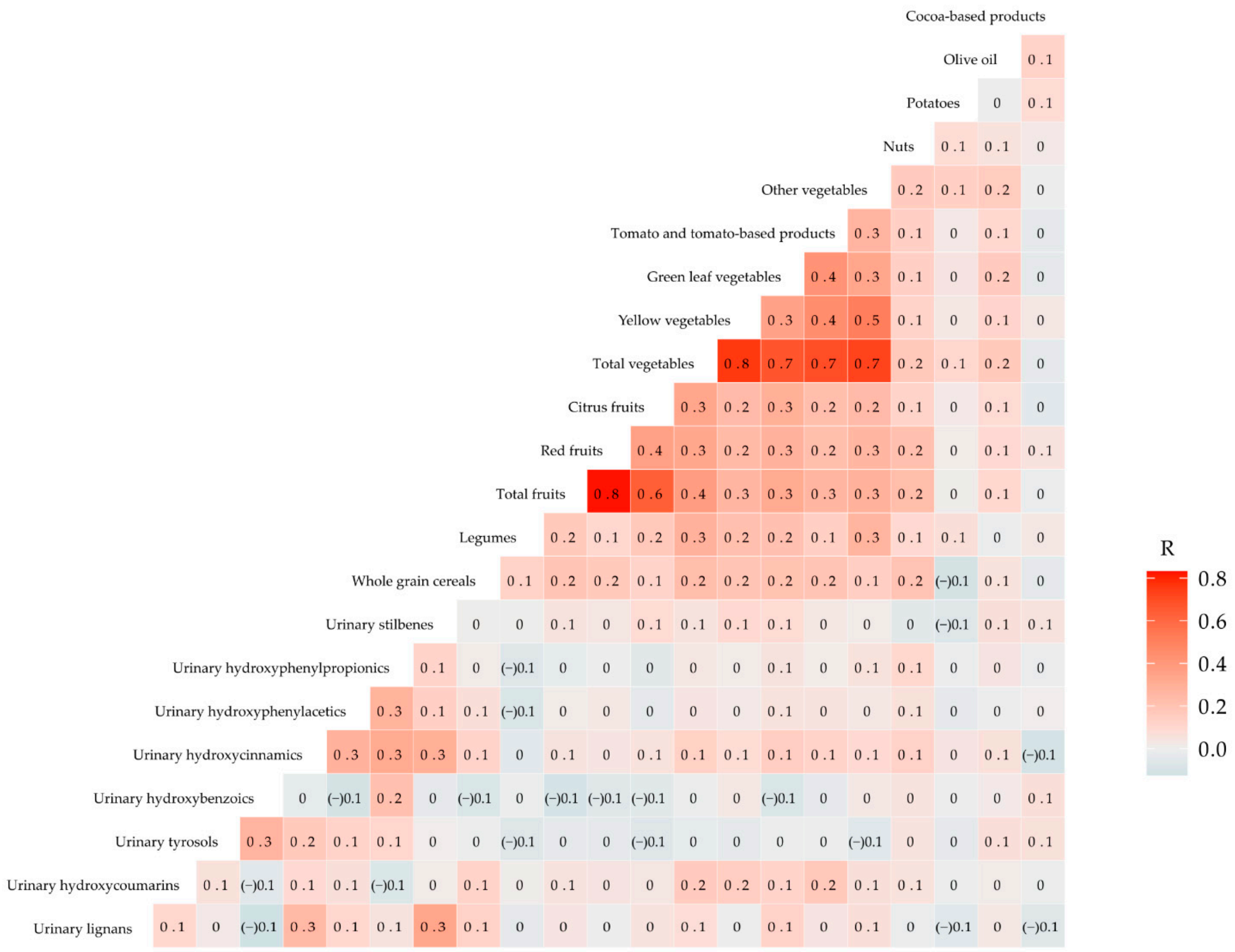

Figure S5. Heatmap of Pearson correlation between subclass of microbial phenolic metabolites and polyphenol-rich food group intake in adolescents.

Table S1. Recovery obtained in Oasis HLB and PRiME HLB.

Phenolic standard	Recovery (%) HLB	Recovery (%) PRiME HLB
Dihydroresveratrol	75%	71%
3,4-Dihydroxyphenylpropionic acid	62%	55%
Enterodiol	69%	76%
Enterolactone	69%	81%
Gallic acid	94%	84%
3-Hydroxybenzoic acid	89%	85%
4-Hydroxybenzoic acid	83%	75%
3′-Hydroxyphenylacetic acid	79%	77%
3-Hydroxytyrosol	86%	78%
3′-Hydroxytyrosol-3′-glucuronide	83%	75%
m-coumaric acid	85%	73%
o-coumaric acid	106%	101%
p-coumaric acid	89%	88%
Protocatechuic acid	98%	88%
Syringic acid	105%	87%
Urolithin-A	96%	58%
Urolithin-B	68%	40%
Vanillic acid	82%	68%

HBL (hydrophilic-lipophilic-balance cartridge). After the selection of Waters Oasis HLB 96-well plates 30 µm (30 mg) as cartridge for the SPE due its higher recovery, four different SPE procedures were tested to obtain higher extraction of phenolic standards. More details are available in "Analytical condition testing before validation HPLC/ESI-LTQ-Orbitrap-HRMS method" from this supplementary data.

Table S2. Characteristics of the validation of the HPLC/ESI-LTQ-Orbitrap-HRMS method: Linearity ranges, coefficient of determination, and low limits of detection and quantification of microbial phenolic metabolites.

Compounds	Rt (min)	Linearity Range (μg/L)	Calibration Curve Equation	R^2	LOD (μg/L)	LOQ (μg/L)
Gallic acid	1.69	2.5–2500	$Y=1875.73x-0.381799x^2+14499.5$	0.9988	0.35	1.16
3-Hydroxytyrosol	2.11	1–1000	$Y = 1582.99x-0.627282x^2+3434.06$	0.9984	0.13	0.43
3′-Hydroxytyrosol-3′-glucuronide	2.11	1–1000	$Y=2284.9x-1.07371x^2+3850.89$	0.9982	0.19	0.64
Protocatechuic acid	2.25	1–1000	$Y = 2456.96x-1.16492x^2+5769.12$	0.9979	0.04	0.13
4-Hydroxybenzoic acid	3.15	1–1000	$Y = 1603.96x-0.383033x^2+5829.04$	0.9992	0.06	0.20
3,4-dihydroxyphenylpropionic acid	3.84	5–5000	$Y = 1584.35x-0.185698x^2+26132.5$	0.9975	0.33	1.11
3-Hydroxybenzoic acid	4.02	1–1000	$Y = 1390.37x-0.660198x^2+937.023$	0.9972	0.21	0.71
3′-Hydroxyphenylacetic acid	4.09	10–100000	$Y = 126.989x-0.00834377x^2-169.942$	0.9979	3.29	10.96
Vanillic acid	4.74	1–1000	$Y = 453.431x-0.023629x^2+304.713$	0.9977	0.18	0.61
Syringic acid	5.63	1–1000	$Y = 1000.29x-0.180888x^2-981.368$	0.9984	0.16	0.53
p-Coumaric acid	5.96	1–1000	$Y = 3148.49x-1.46337x^2+1029.2$	0.9989	0.11	0.36
m-Coumaric acid	6.54	1–1000	$Y = 2498.93x-1.00467x^2+10900.8$	0.9990	0.10	0.32
o-Coumaric acid	7.20	1–1000	$Y = 2340.75x-1.03397x^2-432.812$	0.9988	0.23	0.77
Dihydroresveratrol	8.73	2.50-2500	$Y = 678.148x-0.0970142x^2-1417.62$	0.9992	0.56	1.85
Enterodiol	9.17	1–1000	$Y = 3931.4x-1.75738x^2+5384.13$	0.9979	0.05	0.18
Urolithin A	9.56	2.50–2500	$Y = 484.123x-0.0277372x^2-3593.79$	0.9994	0.61	2.03
Enterolactone	10.80	1–1000	$Y = 4229.75x-1.81222x^2+16770$	0.9956	0.02	0.06
Urolithin B	11.12	1–1000	$Y = 1661.47x-0.424359x^2-3176.34$	0.9990	0.18	0.58

Rt retention time, R^2 coefficient of determination, LOD limit of detection, LOQ limit of quantification.

Table S3. Intra- and inter-day precision and accuracy, matrix effect and recovery results for three concentration levels (high, medium, and low); RSD (%) was calculated for the recovery values for three replicates.

Compound	Concentration (µg/L)	Intra-day assay		Inter-day assay		Matrix effect average (%) (CV)	Recovery Average (%) (CV)
		Precision (RSD%)	Accuracy (%)	Precision (RSD%)	Accuracy (%)		
**Gallic acid**	**12.5**	**7.4**	113.4	58.1	30.8	65.8 (4.6)	70.1 (4.8)
	500	8.8	116.6	13.7	102.6	84.2 (0.7)	88.1 (2.3)
	1915	11.7	87.3	13.3	86.3	88.9 (0.4)	94.0 (1.1)
3-Hydroxytyrosol	5	3.9	99.8	31.1	81.8	116.5 (0.2)	70.1 (1.5)
	200	6.6	101.3	10.1	91.3	88.5 (0.1)	78.1 (6.8)
	766	11.0	90.8	5.2	90.9	94.3 (0.8)	89.6 (4.2)
3′-Hydroxytyrosol-3′-glucuronide	5	4.3	107.8	15.5	92.4	80.4 (1.8)	98.7 (1.7)
	200	6.1	102.9	0.6	102.2	101.8 (1.7)	82.5 (11.2)
	766	8.1	90.4	5.6	90.9	95.1 (0.2)	87.4 (4.6)
Protocatechuic acid	5	5.0	90.2	11.9	86.5	81.5 (0.3)	84.1 (0.6)
	200	3.9	101.4	8.6	106.0	90.5 (1.6)	88.3 (2.7)
	766	7.0	90.9	9.2	96.2	91.8 (0.2)	93.9 (4.2)
4-Hydroxybenzoic acid	5	0.7	99.1	2.6	101.8	125.8 (0.0)	78.1 (0.7)
	200	4.0	96.6	5.2	100.5	101.5 (1.5)	86.7 (3.5)
	766	6.3	97.4	4.9	101.8	99.1 (0.7)	90.5 (1.8)
3,4-dihydroxyphenylpropionic acid	25	8.1	95.1	26.6	13.6	100.9 (3.6)	71.7 (2.7)
	1000	7.2	117.9	7.2	113.5	98.4 (0.9)	92.9 (0.4)
	3830	10.7	81.3	2.2	83.2	95.8 (1.3)	96.4 (2.2)
3-Hydroxybenzoic acid	5	5.3	97.9	4.6	93.1	88.9 (1.4)	104.0 (1.8)
	200	5.7	103.4	3.6	104.4	89.1 (1.4)	95.5 (8.6)
	766	4.3	91.5	3.1	88.5	93.6 (0.1)	96.2 (2.9)
3-Hydroxyphenylacetic acid	50	2.8	94.8	2.0	92.6	91.3 (3.3)	80.2 (1.8)
	2000	3.3	101.1	9.0	111.5	114.0 (0.2)	83.5 (1.3)
	7660	1.7	88.3	8.4	80.9	101.6 (1.7)	94.2 (0.0)
Vanillic acid	5	0.4	94.4	14.3	86.7	110.8 (0.5)	96.0 (4.2)
	200	4.4	94.4	4.4	93.9	91.5 (1.2)	91.1 (6.9)
	766	6.2	97.8	2.3	96.1	96.1 (2.2)	91.9 (10.4)
Syringic acid	5	2.6	110.5	6.5	106.8	77.5 (3.6)	104.5 (0.4)
	200	4.2	99.1	5.2	97.5	93.1 (0.0)	88.8 (4.2)

	766	7.1	97.4	3.5	94.8	91.3 (0.3)	97.6 (2.0)
p-Coumaric acid	5	2.3	99.2	8.6	94.6	83.2 (0.2)	101.4 (0.8)
	200	3.2	101.7	6.9	105.8	93.5 (0.8)	90.8 (3.0)
	766	5.7	89.6	6.0	93.2	101.8 (0.3)	95.5 (1.1)
m-Coumaric acid	5	4.3	98.3	4.3	63.9	110.8 (0.5)	73.8 (0.2)
	200	0.9	100.0	4.7	100.8	94.7 (0.7)	87.4 (1.4)
	766	7.8	93.1	1.2	94.0	94.2 (0.2)	97.6 (3.2)
o-Coumaric acid	5	0.7	95.5	10.5	89.2	75.5 (2.6)	95.0 (0.8)
	200	2.1	101.5	4.6	104.0	87.5 (1.3)	85.8 (0.1)
	766	5.5	92.7	2.2	93.1	89.5 (0.1)	96.2 (0.4)
Dihydroresveratrol	12.5	1.4	105.0	10.2	106.5	60.7 (5.1)	79.1 (1.3)
	500	2.9	100.8	11.7	101.4	66.6 (5.8)	83.0 (4.5)
	1915	8.7	98.3	9.9	100.3	79.0 (1.7)	91.4 (2.8)
Enterodiol	5	5.6	96.9	10.3	87.0	74.1 (6.3)	97.4 (6.9)
	200	3.6	103.2	8.8	98.2	91.6 (0.2)	86.8 (5.3)
	766	10.6	88.0	3.0	85.2	94.2 (0.9)	95.9 (2.1)
Urolithin A	12.5	14.5	112.1	15.4	136.2	7.8 (2.7)	79.9 (3.4)
	500	14.4	92.4	14.6	105.7	17.7 (5.3)	96.4 (3.8)
	1915	8.8	98.8	5.7	101.0	27.7 (8.9)	106.5 (8.6)
Enterolactone	5	4.9	89.8	12.3	100.9	53.9 (2.1)	101.0 (1.1)
	200	4.7	105.6	6.8	110.2	72.5 (0.2)	95.9 (0.2)
	766	9.2	86.2	10.9	90.8	76.2 (3.1)	93.4 (3.6)
Urolithin B	5	5.2	113.1	14.6	117.8	23.7 (9.3)	85.7 (9.0)
	200	4.8	98.3	10.9	108.8	34.0 (4.7)	85.7 (4.3)
	766	10.8	95.3	13.8	105.5	47.2 (5.9)	79.7 (13.2)

RSD relative standard deviation, CV coefficient of variation. 3

Table S4. Identification of microbial phenolic metabolites in urine by HPLC/ESI-LTQ-Orbitrap-HRMS.

Compound	Neutral Molecular Formula	R_t (min)	Ion mass [M-H]-		mDa error	MS^2 fragment ions [M-H]-
			Theoretical	Experimental		
**Lignans - Lignans**						
Enterodiol [a]	C18H22O4	9.17	301.1434	301.1434	0.00	271.1334
Enterodiol glucuronide I (ED)	C24H30O10	7.48	477.1755	477.1751	0.40	459.1652, 301.1442, 175.0244
Enterodiol glucuronide II (ED)	C24H30O10	7.61	477.1755	477.1748	0.70	459.1644, 348.1826, 301.1436, 175.0242
Enterodiol sulfate (ED)	C18H20O7S	7.50	381.0996	381.1005	−0.90	382.1031, 301.1442
Enterolactone [a]	C18H18O4	10.80	297.1121	297.1114	0.70	253.12275, 217.0500, 107.0499
Enterolactone glucuronide (EL)	C24H26O10	8.77	473.1442	473.1434	0.80	455.1339, 343.0944, 297.1128, 175.0243
Enterolactone diglucuronide (EL)	C30H34O16	7.24	649.1763	649.1783	−2.00	473.1469, 297.1135
Enterolactone sulfate (EL)	C18H18O7S	8.57	377.0683	377.0690	−0.70	297.1131
**Phenolic acids-Hydroxybenzoic acids**						
Gallic acid [a]	C7H6O5	1.69	169.0131	169.0141	−1.00	125.0243
Gallic acid glucuronide (GA)	C13H14O11	1.85	345.0452	345.0447	0.50	169.0141, 125.0242
Gallic acid sulfate (GA)	C7H6O8S	1.72	248.9693	248.9700	−0.70	230.0651,204.9810, 169.0141
3- hydroxybenzoic acid [a]	C7H6O3	4.02	137.0233	137.0244	−1.10	93.0342
4-hydroxybenzoic acid [a]	C7H6O3	3.15	137.0233	137.0247	−1.40	93.0342
Hydroxybenzoic acid glucuronide I (HBA)	C13H14O9	2.50	313.0554	313.0548	0.60	175.0244, 137.0241, 93.0342
Hydroxybenzoic acid glucuronide II (HBA)	C13H14O9	2.00	313.0554	313.0551	0.30	295.0821,175.0245, 137.0242, 93.0136
Hydroxybenzoic acid sulfate (HBA)	C7H6O6S	1.89	216.9795	216.9809	−1.40	137.0241, 172.9908, 93.0341
Protocatechuic acid [a]	C7H6O4	2.25	153.0182	153.0197	−1.50	109.029
Protocatechuic acid glucuronide (PCA)	C13H14O10	1.67	329.0503	329.0498	0.50	153.0189, 175.0189, 134.0469
Protocatechuic acid sulfate I (PCA)	C7H6O7S	1.75	232.9744	232.9753	−0.90	153.0191, 188.9856, 109.0290, 96.9596
Protocatechuic acid sulfate II (PCA)	C7H6O7S	1.94	232.9744	232.9754	−1.00	233.9755, 214.9653, 153.0034
Syringic acid [a]	C9H10O5	5.63	197.0444	197.0459	−1.50	153.0557, 121.0295
Syringic acid glucuronide I (SA)	C15H18O11	4.68	373.0765	373.0760	0.50	355.1039, 329.1321, 197.0456, 175.0249
Syringic acid glucuronide II (SA)	C15H18O11	4.88	373.0765	373.0755	1.00	354.0912, 329.1267, 197.0817, 175.0246
Syringic acid sulfate (SA)	C9H10O8S	4.24	277.0006	277.0008	−0.20	197.0455, 182.0219, 167.0715
Vanillic acid [a]	C8H8O4	4.74	167.0339	167.0354	−1.50	123.0450
Vanillic acid glucuronide I (VA)	C14H16O10	2.76	343.0660	343.0651	0.90	175.0245, 167.0347, 123.0448
Vanillic acid glucuronide II (VA)	C14H16O10	2.31	343.0660	343.0650	1.00	175.0245, 167.0348, 131.9496

Vanillic acid sulfate (VA)	C8H8O7S	2.03	246.9910	246.9910	0.00	203.0017, 167.0344, 123.0450
**Phenolic acids - Hydroxycinnamic acids**						
m-coumaric acid [a]	C9H8O3	6.54	163.0390	163.0405	−1.50	119.0500
o-coumaric acid [a]	C9H8O3	7.20	163.0390	163.0403	−1.30	119.0500
p-coumaric acid [a]	C9H8O3	5.96	163.0390	163.0405	−1.30	119.0500
Coumaric acid glucuronide I	C15H16O9	4.77	339.0711	339.0710	0.10	321.0609, 175.0245, 163.0368
Coumaric acid glucuronide II	C15H16O9	5.09	339.0711	339.0707	0.40	295.0391, 175.0247, 163.0400, 119.0500
Coumaric acid glucuronide III	C15H16O9	5.82	339.0711	339.0709	0.20	321.0612, 295.1294, 175.0250, 163.0403, 119.0502
Coumaric acid sulfate I	C9H8O6S	3.53	242.9952	242.9953	−0.10	199.0068, 163.0403, 119.0501, 96.9599
Coumaric acid sulfate II	C9H8O6S	4.27	242.9952	242.9962	−1.00	199.0034, 163.0398, 119.0493
Coumaric acid sulfate III	C9H8O6S	4.74	242.9952	242.9965	−1.30	199.0062, 163.0399, 119.0497
**Phenolic acids - Hydroxyphenylacetic acids**						
3-hydroxyphenylacetic acid [a]	C8H8O3	4.09	151.0390	151.0401	−1.10	107.0208
Hydroxyphenylacetic acid glucuronide (3-HPAA)	C14H16O9	4.8	327.0711	327.0709	0.20	309.0609, 175.0243, 151.0397
Hydroxyphenylacetic acid sulfate (3-HPAA)	C8H8O6S	2.24	230.9952	230.9963	−1.10	151.0399, 108.0208
**Phenolic acids - Hydroxyphenylpropanoic acids**						
3,4-dihydroxyphenylpropionic acid [a]	C9H10O4	3.84	181.0495	181.0508	−1.30	137.0603
Dihydroxyphenylpropionic acid sulfate (3,4-DHPPA)	C9H10O7S	2.52	261.0057	261.0066	−0.90	217.0160, 181.0504, 137.0603
**Stilbenes**						
Dihydroresveratrol [a]	C14H14O3	8.73	229.0865	229.0874	−0.90	185.0819
Dihydroresveratrol sulfate I (DHR)	C14H14O6S	7.07	309.0427	309.0430	−0.30	245.0817, 229.0871, 193.0504, 123.0449
Dihydroresveratrol sulfate II (DHR)	C14H14O6S	7.40	309.0427	309.0430	−0.30	245.0810, 229.0861,175.0235
**Other polyphenols - Hydroxycoumarins**						
Urolithin A [a]	C13H8O4	9.56	227.0339	227.0353	−1.40	183.0047
Urolithin A glucuronide (Uro A)	C19H16O10	6.98	403.0659	403.0666	−0.70	385.0551, 227.0344, 175.0243
Urolithin A diglucuronide (Uro A)	C25H24O16	6.88	579.0981	579.0988	−0.70	403.0688, 227.0352
Urolithin A sulfate (Uro A)	C13H8O7S	7.49	306.9901	306.9906	−0.50	227.0347
Urolithin B [a]	C13H8O3	11.12	211.0390	211.0404	−1.40	167.0499
Urolithin B glucuronide (Uro B)	C19H16O9	8.51	387.0711	387.0713	−0.20	369.1538, 211.0393, 175.0242
**Other polyphenols -Tyrosols**						
3-Hydroxytyrosol [a]	C8H10O3	2.11	153.0546	153.0561	−1.50	123.0452
3′hydroxytyrosol-3′-glucuronide [a]	C14H18O9	2.11	329.0873	329.0878	−0.50	153.056, 123.0452
Hydroxytyrosol sulfate (3-HT)	C8H10O6S	1.82	233.0108	233.0113	−0.50	153.0557, 188.9862, 96.9600

[a]commercial standards, Rt retention time, mDa millidalton of error between the mass found and the accurate mass of each (poly)phenol (absolute value). 3,4-DHPPA 3,4-dihydroxyphenylpropionic acid, 3-HPAA 3-hydroxyphenylacetic acid, 3-HBA 3-hydroxybenzoic acid, 3-HT 3-hydroxytyrosol,

3-HT-G 3-Hydroxytyrosol glucuronide, 4-HBA 4-hydroxybenzoic acid, DHRSV Dihydroresveratrol, ED enterodiol, EL enterolactone, GA gallic acid, PCA protocatechuic acid, SA syringic acid, Uro A urolithin A, Uro B urilithin B, VA vanillic acid. When standards were not available, the aglycone was used for quantification (shown in brackets).

Table S5. Pearson correlation coefficients between microbial phenolic metabolites and dietary polyphenols in adolescents.

Parameter1	Parameter2	R	95% CI		*P*-value
			Low	High	
Urinary lignans	Urinary hydroxycoumarins acids	0.09	0.01	0.17	0.058
Urinary lignans	Urinary tyrosols	0.03	−0.05	0.11	0.654
Urinary lignans	Urinary hydroxybenzoic acids	−0.12	−0.20	−0.04	**0.011**
Urinary lignans	Urinary hydroxycinnamic acids	0.25	0.18	0.33	**<0.001**
Urinary lignans	Urinary hydroxyphenylacetic acids	0.11	0.03	0.19	**0.021**
Urinary lignans	Urinary hydroxyphenylpropionic acids	0.05	−0.03	0.13	0.389
Urinary lignans	Urinary stilbenes	0.34	0.27	0.41	**<0.001**
Urinary lignans	Flavonoid intake	0.02	−0.06	0.11	0.712
Urinary lignans	Phenolic acid intake	0.05	−0.04	0.13	0.468
Urinary lignans	Stilbene intake	0.02	−0.07	0.10	0.797
Urinary lignans	Lignan intake	0.09	0.01	0.18	0.073
Urinary lignans	Tyrosol intake	0.06	−0.02	0.15	0.278
Urinary lignans	Total polyphenol intake	0.04	−0.05	0.12	0.560
Urinary hydroxycoumarin acids	Urinary tyrosols	0.06	−0.02	0.14	0.286
Urinary hydroxycoumarin acids	Urinary hydroxybenzoic acids	−0.04	−0.12	0.04	0.468
Urinary hydroxycoumarin acids	Urinary hydroxycinnamic acids	0.11	0.03	0.19	**0.021**
Urinary hydroxycoumarin acids	Urinary hydroxyphenylacetic acids	0.08	0.00	0.16	0.123
Urinary hydroxycoumarin acids	Urinary hydroxyphenylpropionic acids	−0.04	−0.12	0.04	0.515
Urinary hydroxycoumarin acids	Urinary stilbenes	0.04	−0.04	0.12	0.515
Urinary hydroxycoumarin acids	Flavonoid intake	0.13	0.05	0.21	**0.008**
Urinary hydroxycoumarin acids	Phenolic acids intake	0.08	0.00	0.17	0.123
Urinary hydroxycoumarin acids	Stilbens intake	0.08	−0.01	0.16	0.156
Urinary hydroxycoumarin acids	Lignan intake	0.04	−0.04	0.13	0.497
Urinary hydroxycoumarin acids	Tyrosol intake	0.03	−0.05	0.11	0.608
Urinary hydroxycoumarin acids	Total polyphenol intake	0.13	0.05	0.21	**0.008**
Urinary tyrosols	Urinary hydroxybenzoic acids	0.26	0.19	0.34	**<0.001**
Urinary tyrosols	Urinary hydroxycinnamic acids	0.17	0.09	0.25	**<0.001**
Urinary tyrosols	Urinary hydroxyphenylacetic acids	0.12	0.04	0.19	**0.015**
Urinary tyrosols	Urinary hydroxyphenylpropionic acids	0.13	0.05	0.20	**0.008**
Urinary tyrosols	Urinary stilbenes	0.00	−0.08	0.08	0.952
Urinary tyrosols	Flavonoid intake	−0.03	−0.11	0.06	0.654
Urinary tyrosols	Phenolic acids intake	−0.05	−0.14	0.03	0.389
Urinary tyrosols	Stilbene intake	−0.05	−0.14	0.03	0.389
Urinary tyrosols	Lignan intake	−0.01	−0.09	0.07	0.857
Urinary tyrosols	Tyrosol intake	0.08	−0.01	0.16	0.153
Urinary tyrosols	Total polyphenol intake	−0.02	−0.11	0.06	0.675
Urinary hydroxybenzoic acids	Urinary hydroxycinnamic acids	−0.03	−0.11	0.05	0.585
Urinary hydroxybenzoic acids	Urinary hydroxyphenylacetic acids	−0.11	−0.19	−0.03	**0.016**
Urinary hydroxybenzoic acids	Urinary hydroxyphenylpropionic acids	0.23	0.15	0.30	**<0.001**
Urinary hydroxybenzoic acids	Urinary stilbenes	−0.03	−0.11	0.05	0.585
Urinary hydroxybenzoic acids	Flavonoid intake	−0.01	−0.10	0.07	0.847
Urinary hydroxybenzoic acids	Phenolic acid intake	−0.02	−0.11	0.06	0.712
Urinary hydroxybenzoic acids	Stilbene intake	−0.05	−0.13	0.04	0.468
Urinary hydroxybenzoic acids	Lignans intake	0.02	−0.06	0.10	0.722
Urinary hydroxybenzoic acids	Tyrosol intake	0.05	−0.03	0.14	0.389
Urinary hydroxybenzoic acids	Total polyphenol intake	−0.01	−0.09	0.08	0.927

Urinary hydroxycinnamic acids	Urinary hydroxyphenylacetic acids	0.29	0.21	0.36	**<0.001**
Urinary hydroxycinnamic acids	Urinary hydroxyphenylpropionic acids	0.33	0.25	0.40	**<0.001**
Urinary hydroxycinnamic acids	Urinary stilbenes	0.30	0.22	0.37	**<0.001**
Urinary hydroxycinnamic acids	Flavonoid intake	−0.04	−0.12	0.05	0.560
Urinary hydroxycinnamic acids	Phenolic acid intake	0.05	−0.03	0.13	0.441
Urinary hydroxycinnamic acids	Stilbene intake	0.01	−0.07	0.10	0.843
Urinary hydroxycinnamic acids	Lignan intake	0.09	0.00	0.17	0.092
Urinary hydroxycinnamic acids	Tyrosol intake	0.09	0.01	0.18	0.077
Urinary hydroxycinnamic acids	Total polyphenol intake	−0.02	−0.11	0.06	0.684
Urinary hydroxyphenylacetic acids	Urinary hydroxyphenylpropionic acids	0.27	0.19	0.34	**<0.001**
Urinary hydroxyphenylacetic acids	Urinary stilbenes	0.13	0.05	0.21	**0.005**
Urinary hydroxyphenylacetic acids	Flavonoid intake	0.03	−0.05	0.11	0.608
Urinary hydroxyphenylacetic acids	Phenolic acids intake	0.04	−0.04	0.12	0.515
Urinary hydroxyphenylacetic acids	Stilbene intake	−0.01	−0.09	0.08	0.928
Urinary hydroxyphenylacetic acids	Lignan intake	0.04	−0.04	0.12	0.505
Urinary hydroxyphenylacetic acids	Tyrosol intake	0.00	−0.08	0.09	0.946
Urinary hydroxyphenylacetic acids	Total polyphenol intake	0.03	−0.05	0.12	0.585
Urinary hydroxyphenylpropionic acids	Urinary stilbenes	0.12	0.04	0.20	**0.011**
Urinary hydroxyphenylpropionic acids	Flavonoid intake	−0.05	−0.13	0.04	0.441
Urinary hydroxyphenylpropionic acids	Phenolic acid intake	−0.03	−0.11	0.06	0.663
Urinary hydroxyphenylpropionic acids	Stilbene intake	−0.03	−0.12	0.05	0.585
Urinary hydroxyphenylpropionic acids	Lignan intake	0.04	−0.04	0.13	0.505
Urinary hydroxyphenylpropionic acids	Tyrosol intake	0.02	−0.07	0.10	0.788
Urinary hydroxyphenylpropionic acids	Total polyphenol intake	−0.05	−0.13	0.04	0.441
Urinary stilbenes	Flavonoid intake	0.09	0.01	0.17	0.081
Urinary stilbenes	Phenolic acid intake	0.00	−0.08	0.08	0.991
Urinary stilbenes	Stilbene intake	0.03	−0.05	0.11	0.608
Urinary stilbenes	Lignan intake	−0.07	−0.15	0.02	0.234
Urinary stilbenes	Tyrosol intake	0.01	−0.08	0.09	0.927
Urinary stilbenes	Total polyphenol intake	0.09	0.01	0.18	0.074
Flavonoid intake	Phenolic acid intake	0.51	0.45	0.57	**<0.001**
Flavonoid intake	Stilbene intake	0.60	0.54	0.65	**<0.001**
Flavonoid intake	Lignan intake	0.23	0.15	0.31	**<0.001**
Flavonoid intake	Tyrosol intake	0.05	−0.04	0.13	0.468
Flavonoid intake	Total polyphenol intake	0.98	0.98	0.98	**<0.001**
Phenolic acid intake	Stilbene intake	0.60	0.54	0.65	**<0.001**
Phenolic acid intake	Lignan intake	0.49	0.42	0.55	**<0.001**

Phenolic acid intake	Tyrosol intake	0.16	0.08	0.24	**0.001**
Phenolic acid intake	Total polyphenol intake	0.62	0.56	0.67	**<0.001**
Stilbene intake	Lignan intake	0.28	0.20	0.36	**<0.001**
Stilbene intake	Tyrosol intake	0.07	−0.01	0.15	0.219
Stilbene intake	Total polyphenol intake	0.63	0.57	0.67	**<0.001**
Lignan intake	Tyrosol intake	0.13	0.04	0.21	**0.011**
Lignan intake	Total polyphenol intake	0.29	0.21	0.37	**<0.001**
Tyrosol intake	Total polyphenol intake	0.09	0.01	0.17	0.081

CI confidence interval, R correlation coefficient. False discovery rate method (Benjamini & Hochberg, 1995) was used to adjust *p*-values. *p*-values < 0.5 are in bold.

References

1. Martínez-Huélamo, M.; Tulipani, S.; Jáuregui, O.; Valderas-Martinez, P.; Vallverdú-Queralt, A.; Estruch, R.; Torrado, X.; Lamuela-Raventós, R. Sensitive and Rapid UHPLC-MS/MS for the Analysis of Tomato Phenolics in Human Biological Samples. *Molecules* **2015**, *20*, 20409–20425, doi:10.3390/molecules201119702.
2. Martínez-Huélamo, M.; Tulipani, S.; Torrado, X.; Estruch, R.; Lamuela-Raventós, R.M. Validation of a New LC-MS/MS Method for the Detection and Quantification of Phenolic Metabolites from Tomato Sauce in Biological Samples. *Journal of Agricultural and Food Chemistry* **2012**, *60*, 4542–4549, doi:10.1021/jf205266h.
3. Quifer-Rada, P.; Martínez-Huélamo, M.; Jáuregui, O.; Chiva-Blanch, G.; Estruch, R.; Lamuela-Raventós, R.M. Analytical Condition Setting a Crucial Step in the Quantification of Unstable Polyphenols in Acidic Conditions: Analyzing Prenylflavanoids in Biological Samples by Liquid Chromatography-Electrospray Ionization Triple Quadruple Mass Spectrometry. *Analytical Chemistry* **2013**, *85*, 5547–5554, doi:10.1021/AC4007733
